# Association of Hard Ticks (Ixodidae) Infestation with Milk Production and Udder Health of Extensively Reared Dairy Goats

**DOI:** 10.3390/ani12030354

**Published:** 2022-02-01

**Authors:** Sotiria Vouraki, Athanasios I. Gelasakis, Vasiliki Papanikolopoulou, Elias Papadopoulos, Georgios Arsenos

**Affiliations:** 1 Laboratory of Animal Husbandry, School of Veterinary Medicine, Faculty of Health Sciences, Aristotle University, 54124 Thessaloniki, Greece; vipapani@vet.auth.gr (V.P.); arsenosg@vet.auth.gr (G.A.); 2 Department of Animal Science, School of Animal Biosciences, Agricultural University of Athens, 11855 Athens, Greece; gelasakis@aua.gr; 3 Laboratory of Parasitology and Parasitic Diseases, School of Veterinary Medicine, Faculty of Health Sciences, Aristotle University, 54124 Thessaloniki, Greece; eliaspap@vet.auth.gr

**Keywords:** tick infestation, dairy goats, extensive farming system, milk production, udder health

## Abstract

**Simple Summary:**

Tick infestation and vector-mediated transmission of pathogens thereof challenge the production and health of extensively reared farm animals, causing substantial economic losses and poor welfare. Adverse effects of tick infestation have been documented in cows and sheep. However, relevant studies in goats are scarce. To address this dearth of knowledge, we investigated the association between hard tick infestation, milk production and udder health traits of extensively reared dairy goats in Greece. Tick infestation was significantly associated with impaired udder health, but not with milk yield and quality. Evidence-based tick mitigation strategies in goats are necessary to enhance animal health status and reduce the risk of public health issues deriving from tick-borne pathogen infections.

**Abstract:**

Extensively reared ruminants are seasonally exposed to ticks. Tick-related production losses and health issues have been well documented in cows and sheep but not in goats where relevant literature is scarce. The objective here was to investigate the association of hard tick infestation with milk production and udder health of dairy goats reared extensively. A cross-sectional study was carried out during May and June, in two dairy goat farms. The farms were located in Central and Northern Greece and were representative of typical extensive production systems. A total of 304 goats (*n* = 152 from each farm) were randomly selected. Each goat was examined for presence of hard ticks. Daily milk yield and quality characteristics were recorded. Udder health status was determined by milk somatic cell count (SCC) and total viable count (TVC). Tick infestation prevalence was 28.6%; it was associated with a significant (*p* < 0.001) increase in SCC and TVC (84.0% and 78.6%, respectively). The latter meant that infested goats were 3.7 times more prone to udder health problems (*p* < 0.001). There were not any significant effects (*p* > 0.05) on milk production. Overall, results suggest that control of tick infestation in extensively reared dairy goat herds is important for enhancing health and welfare status.

## 1. Introduction

Ticks are blood-feeding ectoparasites of vertebrate animals. Warm and humid climates favour their survival and activity, and infected pastures facilitate their transmission. As a result, extensively reared ruminants in regions with such climatic conditions are periodically or even permanently challenged by hard tick infestation [1,2]. This pattern is being modified following climate change, a situation which is expected to intensify the problem and broaden the geographical distribution of hard ticks in the future [3,4].

In cattle and sheep farms, tick infestation has been linked to severe monetary losses. For example, an average cost of approximately USD 7.3/animal/year has been estimated in tick-infested dairy cattle [5]. Monetary losses result from indirect (tick-borne diseases) and direct (distress) effects of tick infestation on animal production and health [1,6]. In cattle, tick infestation has been associated with impaired growth, udder health and skin lesions and with reduced milk production [7,8,9,10,11,12,13]. Moreover, in sheep, negative associations with live weight gain, wool production and blood biomarkers (packed cell volume, haemoglobin, and total antioxidant capacity) have been reported [6,14,15].

In extensively reared goats, tick infestation is identified as a major epidemiological issue, especially in tropical and subtropical regions [16]. A high diversity of hard tick species infesting goats has been reported in Middle East and North Africa [17] and a prevalence of *circa* (ca.) 85% found in South Africa [18]. Tick infestation has also been reported in high prevalence in South Asia (ca. 87% and 78–100% in Pakistan and India, respectively [16,19,20]). Moreover, it has been estimated as moderate to high in Eastern Europe (ca. 30% in Southern Greece and 100% in Romania, respectively [21,22]). The impact of tick infestation on goat performance has been accessed in relation to tick-borne diseases such as anaplasmosis, babesiosis and theileriosis [6,23]. Anaplasmosis, caused by *Anaplasma phagocytophilum*, has been found to severely reduce milk yield [23]. The estimated tick-borne disease-related monetary losses in China approximate to USD 2 per goat [24]. However, the direct association between tick infestation and economically important production and health traits remains unknown.

In Greece, dairy goat farming is a significant agricultural activity, with the national herd being the largest in the European Union (ca. 3.6 million [25]). This population is mainly composed of indigenous breeds, namely Eghoria and Skopelos, and reared in extensive farming systems, where goats cover their nutritional demands mostly by exploiting natural pasturelands [26]. In these systems, infestation of goats with ticks mainly of the genus *Rhipichephalus* is commonly observed during the warm months of the year (from May to August) [27,28,29,30], and is expected to escalate in the light of climate change, possibly leading to production losses and health issues. Although chemical control of tick infestation with zero withdrawal time in milk is available [31,32], it is presumed by farmers as a rather costly practice. Moreover, prevention measures, such as rotational grazing and avoiding common pastures shared with other farmers, are rarely implemented. Estimating the possible negative association between tick infestation and goat performance as well as health status could justify the adoption of control and prevention strategies by farmers for improved animal welfare and economically sustainable dairy goat farming.

Therefore, the objective of our study was to investigate the association of hard tick infestation with milk production, and udder health of dairy goats reared under extensive farming systems in Greece.

## 2. Materials and Methods

### 2.1. Herds and Animals

**Figure 1:**
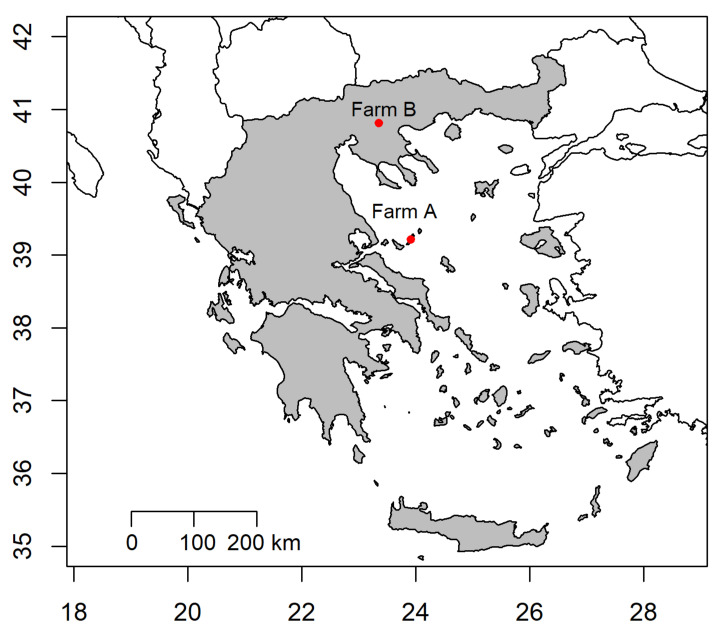
Map of Greece illustrating the regions in which the two studied farms (Farm A and B) were located.

A cross-sectional study was carried out within the framework of Sustainable Organic and Low Input Dairying project (SOLID; 2011–2015) in two representative dairy goat farms with a reported problem of tick infestation, in Central (island of Alonnisos, Farm A) and Northern Greece (Thessaloniki, Farm B, Figure 1) in May and June, respectively. The principal characteristics of the two farms are presented in Table 1. In these farms, a typical grazing scheme throughout the year for traditional goat farming was practiced as described by Gelasakis et al. [19]. In May, the average temperature and humidity in the island of Alonnisos were 20 °C and 68%, respectively, whereas in June the respective values for the area of Thessaloniki were 22 °C and 60% (historical data available at World Weather Online, www.worldweatheronline.com; accessed on 30 December 2021).

A total of 304 adult goats (*n* = 152 in each of the two farms) were randomly selected for the study. Selected goats belonged to two indigenous Greek breeds (Skopelos and Eghoria in Farms A and B, respectively, Table 1).

### 2.2. Data Collection

Animal sampling corresponded to the 5th and 7th month of lactation in Farms A and B, respectively. Prior to milking, all studied goats were thoroughly examined by the same veterinarian for the presence of hard ticks; tick infestation was confirmed when at least 10 ticks were detected. This threshold was defined based on authors’ experience and according to the mean tick infestation severity reported in a previous epidemiological study in Greece [33]. All ticks were carefully removed from the skin of a representative sample of infested goats (20%, *n* = 18) using a pair of forceps and put into a numbered vial containing ethanol 70%. A total of 324 ticks were collected.

Subsequently, a milk sample was collected aseptically from both udder halves in order to be tested for total viable count (TVC, Bactoscan FC). Afterwards, each goat was hand-milked in an individual bucket and the produced milk was weighed. Finally, a milk sample was collected from the milking bucket and used for chemical analyses, which included fat, protein, lactose, and solids-non-fat (SNF) content (MilkoScan FT+) and for somatic cell count (SCC) assessment (Fossomatic FC). Sampling, handling, and analysis of milk samples have been described in detail in previous studies [34,35].

### 2.3. Data Handling

The official A4 method described by the International Committee of Animal Recording [36] was used to estimate daily milk yield. Based on daily milk yield and records of its composition, fat, protein, lactose, and SNF yields were also estimated. Records of SCC and TVC were used to evaluate goat udder health status; goats producing milk with SCC > 10^6^ cells/mL and TVC > 2 × 10^4^ cfu/mL were considered as having impaired udder health status. After quality control, a total of 274, 214 and 303 records of milk composition, SCC and TVC, respectively were considered to be valid for further statistical analysis. The final dataset used for the analysis is available in Dataset S1.

### 2.4. Statistical Analysis

Prevalence of tick infestation and descriptive statistics of all studied traits for tick infested and non-infested goats were calculated within and across farms. Prior to statistical analyses, milk production traits as well as SCC and TVC were logarithmically transformed (natural log) to ensure normality of distribution. Moreover, exploratory data analysis was performed with visual observation of box and whisker plots and count plots (Supplement 1 Figures S1 and S2). Preliminary analyses were performed to identify explanatory variables for all studied traits; the fixed effects of tick infestation, farm and goat age were tested using the forward selection method. Models were tested and compared for goodness of fit using adjusted coefficient of determination, residual standard error, Akaike’s information criterion and Bayesian information criterion; residuals vs. fitted were also plotted (Supplement 1 Table S1 and Figure S3).

According to preliminary analyses, the effect of tick infestation on daily milk, fat, protein, lactose, and SNF yield and milk SCC and TVC was estimated using the following linear model:

(1) $$Y_{ij}{\;=\;\mu +T}_{i}{\;+\;F}_{j}{\;+\;e}_{\mathrm{ij}}$$

where: Y_ij_ is the dependent variable; μ is the overall population mean; T_i_ is the fixed effect of tick infestation (i = 2 levels; 0 = no tick infestation, 1 = tick infestation); F_j_ is the fixed effect of the farm (j = 2 levels; 1= Farm A, 2= Farm B); e_ij_ is the residual error.

The effect of tick infestation on udder health status (binary trait) was analysed with a non-linear model, which included the same effects as model (1) and a logit function for binomial distribution.

Given that breeds and lactation stage were confounded within farms, only the fixed effect of farm was included in the above analyses. All analyses were conducted using R statistical package “stats” [37] and the level of statistical significance was set at *p* = 0.05.

## 3. Results

### 3.1. Descriptive Statistics

**Figure 2:**
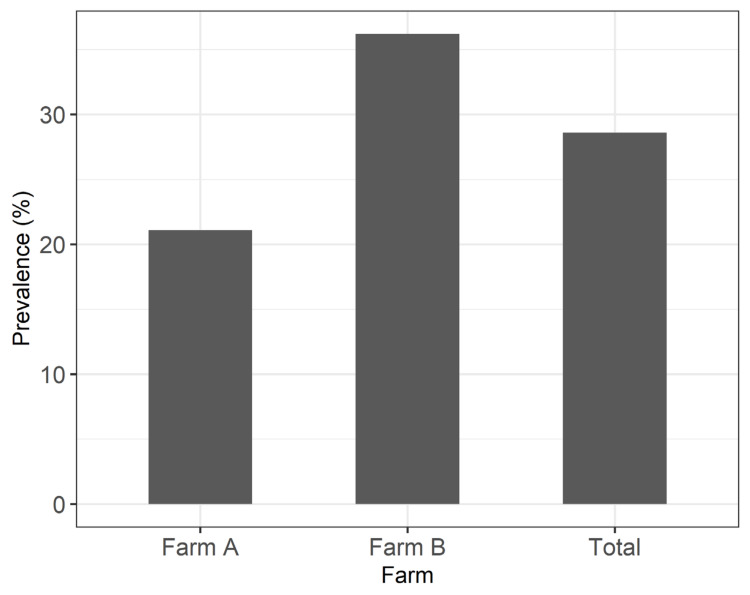
Tick infestation prevalence (%) in the studied farms.

The collected ticks were morphologically identified using standard morphological identification keys [38]. The main (90%, 292/324 ticks) species was *Rhipicephalus sanguineus*, while other minor species (10%, 32/324 ticks) were also detected (*Dermacentor marginatus, Ixodes gibbosus, Rhipicephalus bursa*, and *Ixodes ricinus*). Prevalence of tick infestation is presented in Figure 2. Across farms, a prevalence of 28.6% (87/304 goats) was found. Between farms, the highest prevalence was reported in Farm B (36.2% vs. 21.1% in Farm A).

**Figure 3:**
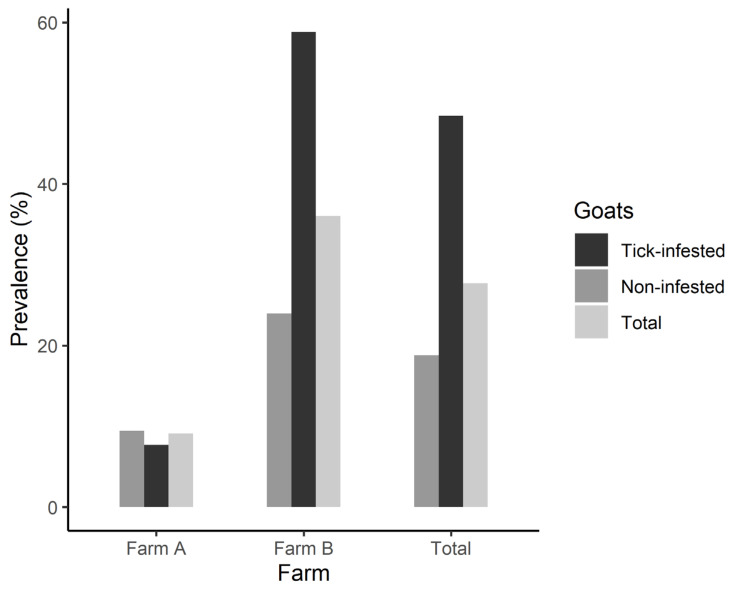
Impaired udder health status prevalence in the studied farms for tick-infested, non-infested and all (total) studied goats.

Descriptive statistics of all continuous studied goat traits and prevalence of impaired udder health status for tick-infested and non-infested goats are presented in Table 2 and Figure 3, respectively. Across farms, the average daily milk yield and milk components were lower in infested compared to non-infested goats, whereas milk SCC and TVC were higher (Table 1). Moreover, a higher prevalence of impaired udder health status was found in infested goats (48.4%, 31/64 goats) compared to non-infested ones (18.8%, 28/149 goats).

### 3.2. Effect of Tick Infestation on Goat Productivity and Udder Health

The effects of tick infestation on milk production and udder health traits are presented in Table 3; significant effects (*p* < 0.01) were found on udder health traits. Specifically, tick infestation resulted in an increase in SCC by 84.0% (±19.72%, *p* < 0.001) and TVC by 78.6% (±17.59%, *p* < 0.001). Moreover, infested goats were 3.65 times more likely to have impaired udder health status (*p* < 0.001) compared to non-infested ones. No significant effects were reported on milk production traits (*p* > 0.05).

## 4. Discussion

To the best of our knowledge this is the first study of the possible association of tick infestation with milk production and udder health traits of dairy goats reared in extensive farming systems. Tick infestation was associated with impaired udder health status, but not with milk yield and quality.

In cattle, the impact of tick infestation on animal performance has been mostly investigated under experimental conditions involving artificial infestations [8,9,10,39]. This allows for a true control group avoiding possible confounding effects. However, it could lead to overestimations compared to the situation on field [11]. In our study, this impact was investigated in extensively reared goats under natural tick infestation conditions in order to reveal possible associations that could be attributable to tick-infestation during the ticks’ highest activity season. Previous studies have shown that ticks of the genus *Rhipicephalus*, which are the most prevalent hard ticks in goat herds in Greece (ca. 90%, [30]), are more active during the warm period of the year and especially from May to July/August [27,28]. For this reason, in our study, sampling was performed within this period and specifically, in May and June for Farms A and B, respectively; this decision was also supported by past observations of farmers who reported a systematic tick infestation problem during these months.

The reported tick infestation prevalence in the studied farms was ca. 29% and in agreement with the one reported by Dimanopoulou et al. [21] in areas of Southern Greece (ca. 30%). However, it was lower compared to the estimates in Romania, South Asia, and South Africa (ca. 85−100% [16,18,19,20,22]). Between the two studied farms, the prevalence was relatively higher in Farm B located in the area of Thessaloniki (Northern Greece). Given that the climatic conditions (temperature and humidity) in the two studied areas were similar at the time of tick infestation assessment (historical data available at World Weather Online, www.worldweatheronline.com; accessed on 30 December 2021), this difference might be associated with diverse vegetation patterns, livestock stocking rate, and soil microclimate (soil surface temperature and relative humidity, [4]) and/or with different host resistance levels.

The impact of tick infestation on milk production has been widely studied in dairy cows. In Australia and Africa, infestation with ticks of the genus *Rhipicephalus* spp. was found to result in significant loss of daily milk yield (ca. 9 mL loss per engorged tick) in Holstein–Friesian and Sanga cattle, respectively [8,10]. Moreover, in Brazil, tick infestation was associated with a significant reduction in the total lactation milk yield of Holstein × Zebu cows (ca. 90 litres/cow, [13]). Such findings have been attributed to the loss of appetite resulting in reduced feed intake and, consequently, energy loss ([10]. However, no significant associations were reported in the studies of Norval et al. [9,39] for dairy cattle infested with *Rhipicephalus* spp. and *Amblyomma* spp. ticks. The latter findings are in accordance with those of the present study for dairy goats, where the association with daily milk yield was negative but not statistically significant. Moreover, no other significant effects on milk components (fat, protein, lactose and SNF) yield were found, which is supported by the findings of Jonsson et al. [10] regarding fat and protein yield, and content in the milk of tick-infested goats.

According to research in cattle, tick infestation severity could be a source of variation dictating the significance of effects on productivity [10,11]. In our study, such an investigation was not performed since counting of the total number of ticks engorged in each animal was not feasible and would cause unnecessary distress in the animals. Moreover, the number of ticks infesting an animal is a dynamic situation not easily described under a cross-sectional epidemiological study-design. In a previous epidemiological study in Greece, tick infestation severity in May and June ranged from 1 to 21 engorged ticks per goat [33]. Given this variability, it could be speculated that significant adverse effects might occur in cases of severe tick infestation. This is further supported by the fact that, although not statistically significant, a decrease in all studied milk production traits was reported in infested compared to non-infested goats. Therefore, the effect on goat productivity should be further studied after quantifying infestation to account it as an infestation severity index; in any case, a prospective study-design is warranted.

Significant effects of tick infestation were found on udder health; infested goats had increased milk SCC and TVC (ca. 80% increase, in each case) and were more likely (ca. 4 times) to have impaired udder health status. In dairy cows, Jonsson et al. [10] found no significant effect of tick infestation on milk SCC. However, in other studies [9,12], a significant adverse association with SCC as well as the incidence of mastitis has been reported, in consistence with the results of our study. Such an adverse effect on udder health could be possibly explained by an overall immunosuppressive impact of tick infestation and/or increased oxidative stress. Iqbal et al. [40] reported a significant reduction in the number of lymphocytes in goats infested with ectoparasites including ticks. Moreover, in cows, tick infestation has been shown to reduce the percentage of T lymphocytes and, in a lesser extent, B lymphocytes and the antibody response to specific protein antigens [7] reaffirming immunosuppression potential. Furthermore, in tick-infested sheep, an increase of serum total antioxidant capacity has been reported, which is likely to predispose to several health disorders [15]. Further relevant research in goats could help towards understanding the underlying mechanisms and sufficiently elucidate the adverse association between tick infestation and goat udder health status.

In the present study, tick infestation was defined by taking into account ticks attached to any part of the body. However, the reported adverse association with goat udder health status could have also resulted from the inflammation and transmission of mastitis-related pathogens from ticks specifically attached to the udder. In the study of Moges et al. [12], the presence of ticks and/or lesions on the udder of dairy cows was found to be a risk factor of mastitis. *Staphylococcus aureus*, the most common pathogen among coagulase-positive staphylococci leading to clinical and subclinical mastitis [41], has been isolated from ticks of the genus *Rhipichephalus* spp. that infested sheep and cattle in Iraq and Texas [42,43]. Moreover, in the study of Andreotti et al. [43], coagulase-negative staphylococci and *Streptococcus* spp. have also been isolated from ticks of the same genus. In addition, a variety of Gram-negative bacteria, including *Escherichia coli*, *Salmonella* spp., *Klebsiella* spp., *Serratia* spp., and *Enterobacter* spp., have been isolated from *Rhipichephalus* spp. ticks in Turkey and Iraq [42,44]. All the above bacteria have been also isolated from the milk of extensively reared dairy goats with subclinical mastitis in Greece [33]. Future studies investigating microbiological cultures from the milk of dairy goats infested with ticks attached to the udder skin could help to identify tick-borne bacterial pathogens and verify their association with udder health; valuable information could also be provided by studying the tick microbiome.

In our study, goats were considered to have impaired udder health status when producing milk with SCC > 10^6^ cells/mL and TVC > 2 × 10^4^ cfu/mL. In the study of Gelasakis et al. [34], 78% of goat milk samples with SCC and TVC above these limits were found to have positive microbiological cultures, therefore suggesting a strong association with subclinical mastitis. Subclinical mastitis is known to cause major financial losses for small ruminant farmers due to increased treatment and replacement costs and adverse effects on milk production [45,46]. Specifically, in extensive dairy goat herds in Greece, subclinical mastitis has been associated with reduced daily milk yield (by 5.7%) as well as fat and lactose yield [34,35]. Therefore, although no direct effects of tick infestation on milk production were reported in our study, indirect effects and, hence, economic losses due to impaired goat udder health status could be assumed.

Based on our findings and previous knowledge on production losses, mortality rates, and veterinary treatment costs associated with tick-borne diseases [15,23,24], tick infestation could be considered an issue of substantial economic importance for extensive goat farming systems. This importance may be even greater in the near future given that climate change is expected to impact the population and transmission dynamics, seasonal activity, and abundance of ticks [3,4,47]. In this regard, prevention and control measures against tick infestation could help towards increasing the economic sustainability of the sector and the health and welfare status of the animals. In addition to health and welfare-related issues underpinning the necessity of tick mitigation strategies, further research taking into account tick infestation severity could further help to increase their adoption by estimating an economic threshold above which chemical control of tick infestation would be cost-effective [11]. Moreover, adoption of tick control measures is expected to be beneficial towards a one health perspective considering that ticks are vectors of many zoonoses [48].

## 5. Conclusions

According to the present study, tick infestation significantly contributes to udder health problems, though it does not seem to affect milk production of dairy goats reared in extensive farming systems in Greece. Adoption of prevention and available control measures to reduce tick infestation prevalence in dairy goat herds is expected to improve goats’ udder health status and, hence, be also beneficial in terms of production and financial sustainability. Further research taking into account tick infestation severity is recommended for assessing potential adverse effects on goat productivity and for establishing an economic threshold for tick infestation control.

## Figures and Tables

**Table 1:** Principal characteristics of the two goat farms that participated in the study.

Characteristic	Farm A	Farm B
Breed	Skopelos	Eghoria
Number of adult goats	250	1200
Number of bucks	20	85
Number of yearlings	70	215
Goat replacement rate (%)	15	15
Buck replacement rate (%)	25	30
Age of yearlings at first mating (months)	9	7
Milk production (kg/goat/lactation period)	280	180
Kidding season	December	November
Ectoparasitic treatment	ivermectin	ivermectin
Last ectoparasitic treatment (months ago)	7	9
Sampling month	May	June
Grazing duration (hours/day)	10	6
Type of pastureland	grassland/shrubland/woodland	grassland/shrubland/woodland

**Table 2:** Descriptive statistics for goat traits studied in Farms A and B for tick-infested, non-infested and all (total) studied goats.

		Farm A		Farm B		Total	
Trait	Goats	*N*	Mean (±SD ^1^)	*N*	Mean (±SD ^1^)	*N*	Mean (±SD ^1^)
Daily milk yield (g)	Tick-infested	32	1156.7 (419.82)	55	659.0 (173.61)	87	842.1 (375.14)
	Non-infested	120	1063.2 (348.81)	97	742.0 (186.53)	217	919.6 (328.81)
	Total	152	1082.9 (365.42)	152	712.0 (185.74)	304	897.4 (343.87)
Daily fat yield (g)	Tick-infested	26	57.1 (17.95)	52	31.6 (8.54)	78	40.1 (17.30)
	Non-infested	99	55.2 (15.53)	97	35.0 (8.75)	196	45.2 (16.18)
	Total	125	55.6 (16.01)	149	33.8 (8.80)	274	43.8 (16.63)
Daily protein yield (g)	Tick-infested	26	44.6 (14.82)	52	24.4 (5.84)	78	31.2 (13.62)
	Non-infested	99	39.7 (10.89)	97	26.1 (6.46)	196	33.0 (11.26)
	Total	125	40.7 (11.92)	149	25.5 (6.28)	274	32.5 (11.98)
Daily lactose yield (g)	Tick-infested	26	49.4 (20.52)	52	28.2 (7.18)	78	35.3 (16.48)
	Non-infested	99	43.9 (13.89)	97	31.4 (7.96)	196	37.7 (12.93)
	Total	125	45.0 (15.57)	149	30.3 (7.82)	274	37.0 (14.04)
Daily SNF ^2^ yield (g)	Tick-infested	26	104.7 (39.22)	52	58.7 (14.01)	78	74.0 (33.23)
	Non-infested	99	93.2 (27.47)	97	64.2 (15.83)	196	78.8 (26.72)
	Total	125	95.6 (30.47)	149	62.3 (15.40)	274	77.5 (28.74)
Milk SCC ^3^ (×10^3^ cells/mL)	Tick-infested	13	1455.1 (2052.42)	52	3238.8 (3736.53)	65	2882.0 (3525.98)
	Non-infested	53	1723.4 (3940.92)	96	1377.8 (1693.78)	149	1500.7 (2706.63)
	Total	66	1670.6 (3635.10)	148	2031.7 (2737.25)	214	1920.3 (3038.30)
Milk TVC ^4^ (×10^3^ cfu/mL)	Tick-infested	32	10.2 (8.17)	54	323.9 (581.77)	86	207.2 (484.09)
	Non-infested	120	53.1 (265.77)	97	87.7 (251.11)	217	68.6 (259.30)
	Total	152	44.1 (236.62)	151	172.2 (415.75)	303	107.9 (343.46)

^1^ SD = standard deviation ^2^ SNF = solids-non-fat ^3^ SCC = somatic cell count ^4^ TVC = total viable count.

**Table 3:** Effects (β-coefficients, standard errors) of tick infestation on milk production and udder health traits.

Trait	β-Coefficient	SE ^1^	*p*-Value
Daily milk yield (g, ln)	−0.04	0.040	0.333
Daily fat yield (g, ln)	−0.06	0.039	0.136
Daily protein yield (g, ln)	0.00	0.037	0.984
Daily lactose yield (g, ln)	−0.03	0.042	0.433
Daily SNF ^2^ yield (g, ln)	−0.02	0.039	0.659
Milk SCC ^3^ (cells/mL, ln)	0.61	0.176	<0.001
Milk TVC ^4^ (cfu/mL, ln)	0.58	0.162	<0.001
Udder health status (odds ratio)	3.65	1.24	<0.001

^1^ SE = standard error ^2^ SNF = solids-non-fat ^3^ SCC = somatic cell count ^4^ TVC = total viable count.

## Data Availability

Data presented in this study is contained within the article and Supplementary Materials (Dataset S1 and Supplement 1).

## Supplementary Materials

The following are available online at https://www.mdpi.com/article/10.3390/ani12030354/s1, Dataset S1: Total dataset of goat traits used for the analyses. Figure S1: Distribution of goat age in the studied Farms A and B (count plot); Figure S2: Relationship (A-G: box and whisker plots and H: count plot) of milk production and udder health traits with tick infestation; Figure S3: Residuals vs fitted plots for linear models used to analyse the association of tick infestation with milk production and udder health traits (SNF = solids-non-fat; SCC = somatic cell count; TVC = total viable count); Table S1: Comparison of goodness of fit of linear and non-linear models tested for estimating the effect of tick infestation on milk production and udder health goat traits.
