# Supplementary material for: Association of Hard Ticks (Ixodidae) Infestation with Milk Production and Udder Health of Extensively Reared Dairy Goats
Source: Animals (Basel). 2022 Feb 1;12(3):354. doi: 10.3390/ani12030354 (PMC8833764; doi:10.3390/ani12030354)
Supplement: Supplementary file 1 [file animals-12-00354-s001.zip › Supplement 1.pdf]

Association of hard ticks (Ixodidae) infestation with milk production and udder health of extensively reared dairy goats

S. Vouraki, A.I. Gelasakis, V. Papanikolopoulou, E. Papadopoulos and G. Arsenos

Supplement 1

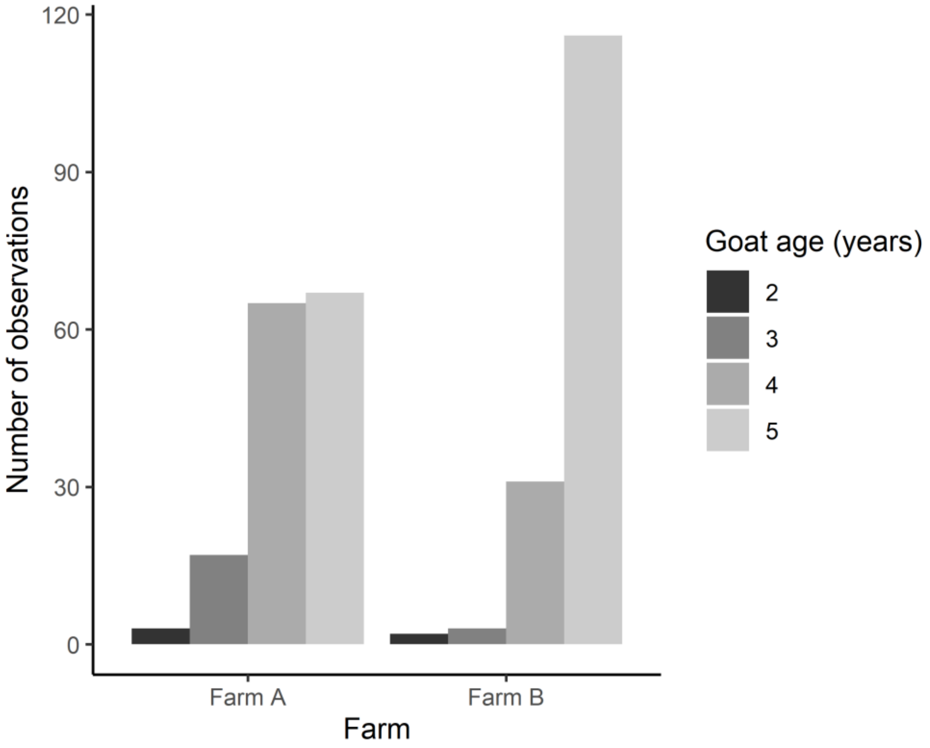

Figure S1. Distribution of goat age in the studied Farms A and B (count plot).

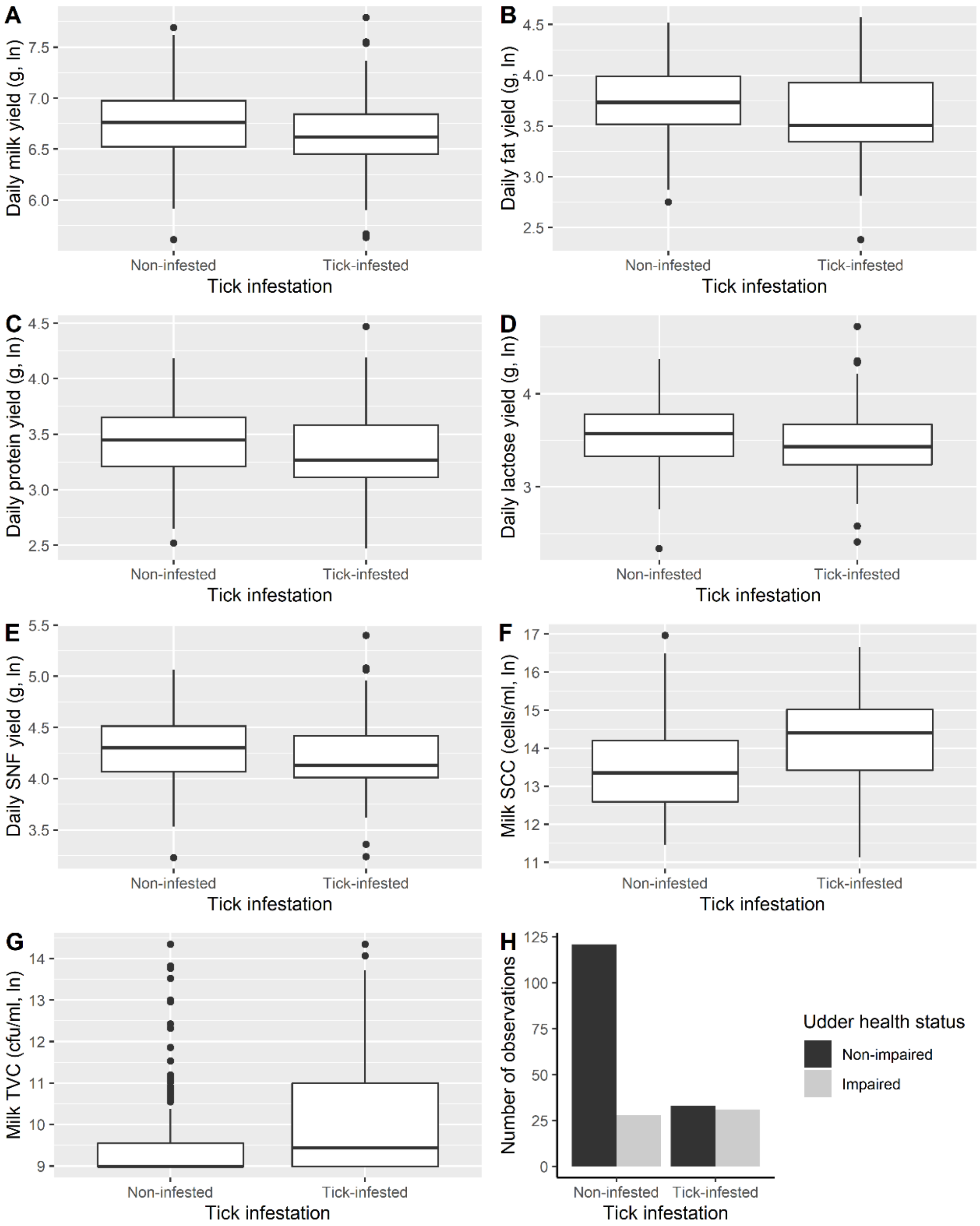

Figure S2. Relationship (A-G: box and whisker plots and H: count plot) of milk production and udder health traits with tick infestation.

Table S1. Comparison of goodness of fit of linear and non-linear models tested for estimating the effect of tick infestation on milk production and udder health goat traits.

Response variable	Model	Adjusted R^2	RSE	AIC	BIC
Daily milk yield (g, ln)	$y_i = \mu + T_i + e_i$ [1]	0,016	0,367	258	269
	$y_{ij} = \mu + T_i + F_j + e_{ij}$ [2]	0,290	0,312	159	174
	$y_{ijm} = \mu + T_i + F_j + A_m + e_{ijm}$ [3]	0,292	0,311	161	187
Daily fat yield (g, ln)	$y_i = \mu + T_i + e_i$ [1]	0,025	0,374	242	253
	$y_{ij} = \mu + T_i + F_j + e_{ij}$ [2]	0,416	0,289	103	117
	$y_{ijm} = \mu + T_i + F_j + A_m + e_{ijm}$ [3]	0,416	0,289	106	131
Daily protein yield (g, ln)	$y_i = \mu + T_i + e_i$ [1]	0,006	0,353	211	222
	$y_{ij} = \mu + T_i + F_j + e_{ij}$ [2]	0,408	0,273	70,2	84,7
	$y_{ijm} = \mu + T_i + F_j + A_m + e_{ijm}$ [3]	0,409	0,272	72,6	97,9
Daily lactose yield (g, ln)	$y_i = \mu + T_i + e_i$ [1]	0,011	0,36	222	233
	$y_{ij} = \mu + T_i + F_j + e_{ij}$ [2]	0,256	0,313	145	160
	$y_{ijm} = \mu + T_i + F_j + A_m + e_{ijm}$ [3]	0,257	0,312	148	173
Daily SNF yield (g, ln)	$y_i = \mu + T_i + e_i$ [1]	0,009	0,352	210	220
	$y_{ij} = \mu + T_i + F_j + e_{ij}$ [2]	0,328	0,29	104	118
	$y_{ijm} = \mu + T_i + F_j + A_m + e_{ijm}$ [3]	0,330	0,29	106	132
Milk SCC (cells/ml, ln)	$y_i = \mu + T_i + e_i$ [1]	0,063	1,2	688	698
	$y_{ij} = \mu + T_i + F_j + e_{ij}$ [2]	0,103	1,17	680	693
	$y_{ijm} = \mu + T_i + F_j + A_m + e_{ijm}$ [3]	0,115	1,16	680	703
Milk TVC (cfu/ml, ln)	$y_i = \mu + T_i + e_i$ [1]	0,059	1,36	1052	1063
	$y_{ij} = \mu + T_i + F_j + e_{ij}$ [2]	0,208	1,25	1001	1015
	$y_{ijm} = \mu + T_i + F_j + A_m + e_{ijm}$ [3]	0,209	1,25	1003	1029
Udder health status (0-1)	$y_i = \mu + T_i + e_i$ [1]	0.075 (Pseudo $R^{2)}$	NA	237	NA
	$y_{ij} = \mu + T_i + F_j + e_{ij}$ [2]	0.134 (Pseudo R^2)	NA	224	NA
	$y_{ijm} = \mu + T_i + F_j + A_m + e_{ijm}$ [3]	0.142 (Pseudo R^2)	NA	224	NA

RSE = residual standard error; AIC = Akaike's information criterion; BIC = Bayesian information criterion; SNF = solids-non-fat; SCC = somatic cell count; TVC = total viable count; μ = overall population mean; T_i = fixed effect of tick infestation (i = 2 levels; 0 = no tick infestation, 1 = tick infestation); F_j = fixed effect of farm (j = 2 levels; 1= Farm A, 2= Farm B); A_m = fixed effect of goat age in years (m= 4 levels; 1= two years, 2= three years, 3 = four years; 4= ≥five years); e = residual error.

All final analyses were performed using model [2].

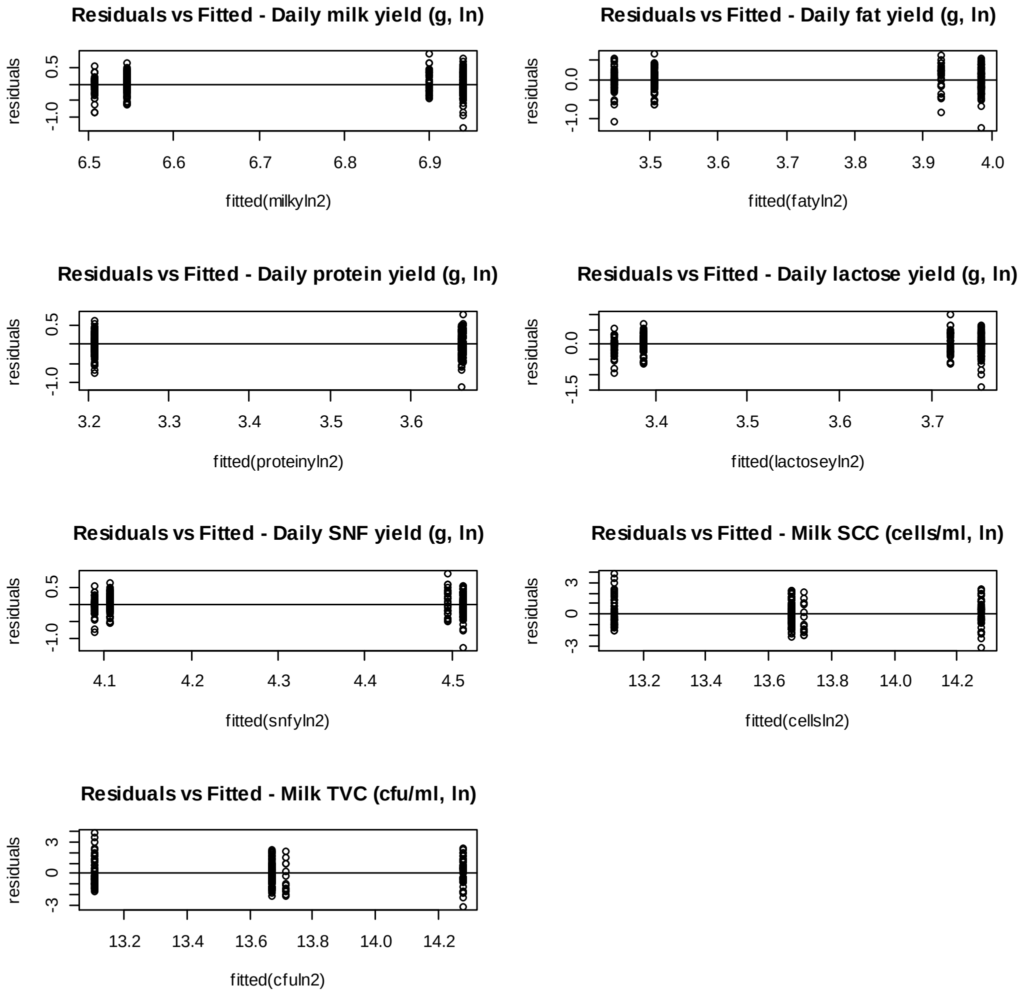

Figure S3. Residuals vs fitted plots for linear models used to analyse the association of tick infestation with milk production and udder health traits (SNF= solids-non-fat; SCC = somatic cell count; TVC = total viable count).
